# (4*S*,5*S*)-2-(2-Bromo­phen­yl)-1,3-dioxolane-4,5-dicarboxamide

**DOI:** 10.1107/S1600536809010009

**Published:** 2009-03-25

**Authors:** Hua-quan Liu, De-Cai Wang, Wei Xu, Zheng Yang, Tao Gai

**Affiliations:** a State Key Laboratory of Materials-Oriented Chemical Engineering, College of Life Science and Pharmaceutical Engineering, Nanjing University of Technology, Xinmofan Road No. 5 Nanjing, Nanjing 210009, People’s Republic of China

## Abstract

The asymmetric unit of the title compound, C_11_H_11_BrN_2_O_4_, contains two crystallographically independent mol­ecules in which the bromo­phenyl rings are oriented at dihedral angles of 39.28 (3)°. The dioxolane rings adopt envelope conformations. Intra­molecular N—H⋯O hydrogen bonds result in the formation of four five-membered rings, having planar and envelope conformations. In the crystal structure, inter­molecular N—H⋯O hydrogen bonds link mol­ecules into chains along the *b* axis, forming *R*_2_^2^(8) ring motifs.

## Related literature

For the use of similar compounds in the synthesis of platinum based anti-tumour agents and in organic syntheses, see: Kim *et al.* (1994 ▶); Pandey *et al.* (1997 ▶). For bond-length data, see: Allen *et al.* (1987 ▶). For ring motifs, see: Bernstein *et al.* (1995 ▶).
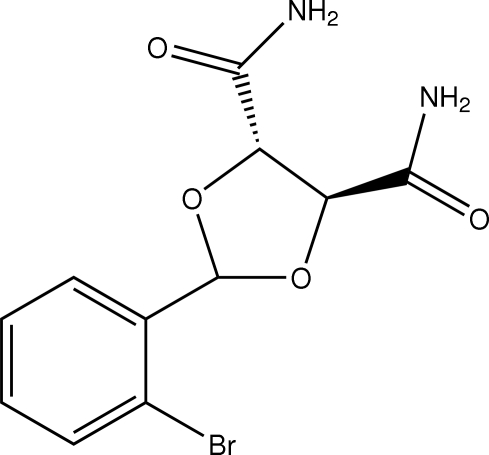


## Experimental

### 

#### Crystal data

C_11_H_11_BrN_2_O_4_
*M* *_r_* = 315.12
Monoclinic, 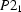

*a* = 9.4150 (19) Å
*b* = 14.458 (3) Å
*c* = 9.6170 (19) Å
β = 111.14 (3)°
*V* = 1221.0 (5) Å^3^
*Z* = 4
Mo *K*α radiation
μ = 3.38 mm^−1^
*T* = 294 K
0.20 × 0.10 × 0.10 mm

#### Data collection

Enraf–Nonius CAD-4 diffractometer
Absorption correction: ψ scan (North *et al.*, 1968 ▶) *T*_min_ = 0.552, *T*_max_ = 0.729
4504 measured reflections
4256 independent reflections
2669 reflections with *I* > 2σ(*I*)
*R*_int_ = 0.061
3 standard reflections frequency: 120 min intensity decay: 1%

#### Refinement

*R*[*F* ^2^ > 2σ(*F* ^2^)] = 0.063
*wR*(*F* ^2^) = 0.181
*S* = 1.00
4256 reflections
325 parameters
H-atom parameters constrained
Δρ_max_ = 0.54 e Å^−3^
Δρ_min_ = −0.82 e Å^−3^
Absolute structure: Flack (1983 ▶), 1758 Friedel pairs
Flack parameter: 0.00 (2)

### 

Data collection: *CAD-4 Software* (Enraf–Nonius, 1989 ▶); cell refinement: *CAD-4 Software*; data reduction: *XCAD4* (Harms & Wocadlo, 1995 ▶); program(s) used to solve structure: *SHELXS97* (Sheldrick, 2008 ▶); program(s) used to refine structure: *SHELXL97* (Sheldrick, 2008 ▶); molecular graphics: *ORTEP-3 for Windows* (Farrugia, 1997 ▶) and *PLATON* (Spek, 2009 ▶); software used to prepare material for publication: *SHELXTL* (Sheldrick, 2008 ▶).

## Supplementary Material

Crystal structure: contains datablocks global, I. DOI: 10.1107/S1600536809010009/hk2643sup1.cif

Structure factors: contains datablocks I. DOI: 10.1107/S1600536809010009/hk2643Isup2.hkl

Additional supplementary materials:  crystallographic information; 3D view; checkCIF report

## Figures and Tables

**Table 1:** Hydrogen-bond geometry (Å, °)

*D*—H⋯*A*	*D*—H	H⋯*A*	*D*⋯*A*	*D*—H⋯*A*
N1—H1*B*⋯O1	0.86	2.25	2.657 (12)	109
N2—H2*B*⋯O2	0.86	2.27	2.660 (12)	107
N3—H3*B*⋯O8^i^	0.86	2.28	3.123 (14)	167
N3—H3*C*⋯O5	0.86	2.32	2.684 (14)	106
N4—H4*B*⋯O7^ii^	0.86	2.05	2.876 (11)	160
N4—H4*C*⋯O6	0.86	2.26	2.672 (11)	110

Symmetry codes: (i) 
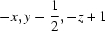
; (ii) 
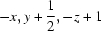
.

**Fig. 1.**
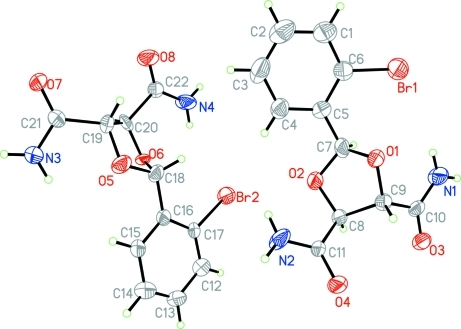
The molecular structure of the title molecule, with the atom-numbering scheme. Displacement ellipsoids are drawn at the 30% probability level.

**Fig. 2.**
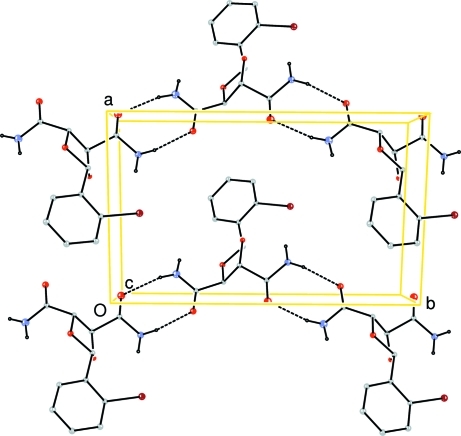
A partial packing diagram of the title compound. Hydrogen bonds are shown as dashed lines.

## supplementary crystallographic information

### Comment

Antitumor platinum drug is one kind of the most effective anticancer agents currently available. (2S,3S)-Diethyl 2,3-O-alkyltartrate analogues are starting materials for the syntheses of platinum complexes with antitumor activity (Kim *et al.*,  1994), and are also important intermediates in organic syntheses (Pandey *et al.*,  1997). As part of our studies on the syntheses and characterizations of these compounds, we have synthesized the title compound and reported herein its crystal structure.

The asymmetric unit of the title compound contains two crystallographically independent molecules (Fig. 1), in which the bond lengths (Allen *et al.*, 1987) and angles are within normal ranges. Rings A (C1-C6) and A' (C12-C17) are, of course, planar and they are oriented at a dihedral angle of A/A' = 39.28 (3)°. Rings B (O1/O2/C7-C9) and B' (O5/O6/C18-C20) adopt envelope conformations with C7 and C18 atoms displaced by 0.503 (3) and -0.589 (3) Å from the planes of the other ring atoms, rspectively. The intramolecular N-H···O hydrogen bonds (Table 1) result in the formations of four five-membered rings: C (O1/N1/C9/C10/H1B), D (O2/N2/C8/C11/H2B) and C' (O5/N3/C19/C21/H3C), D' (O6/N4/C20/C22/H4C). Ring D is planar, while rings C, C' and D' have envelope conformations with atoms O1, O5 and O6 displaced by 0.223 (3), -0.530 (3) and -0.304 (3) Å, respectively, from the planes of the other ring atoms.

In the crystal structure, intermolecular N-H···O hydrogen bonds (Table 1) link the molecules into chains along the b-axis, forming R_2_^2^(8) ring motifs (Fig. 2) (Bernstein *et al.*,  1995). in which they may be effective in the stabilization of the structure.

### Experimental

For the preparation of the title compound, 2-bromobenzaldehyde (300 mg, 1.62 mmol), (2S,3S)-diethyltartrate (434 mg, 2.11 mmol) and cyclohexane (10 ml) were placed in a round-bottomed flask, and 4-methylbenzenesulfonic acid (30 mg) was added. The flask was fitted with a water-distributor. The mixture was heated under reflux for 3 h. The reaction mixture was cooled to room temperature, and then transferred into a separatory funnel, washed with water (200 ml) and extracted with acetate (200 ml). The organic phase was distilled under pressure, and the residual was dissolved in anhydrous ethanol (50 ml). Then, a current of dry ammonia was passed through the reaction mixture at room temperature for about 4 h. The reaction mixture was then added dropwise to a vigorously stirred water (600 ml). The resulting colorless precipitate was obtained by filtration and dried in vacuo (Kim *et al.*,  1994). Crystals suitable for X-ray analysis were obtained by slow evaporation of a methanol solution after two weeks.

### Refinement

H atoms were positioned geometrically, with N-H = 0.86 Å (for NH_2_) and C-H = 0.93 and 0.98 Å for aromatic and methine H, respectively, and constrained to ride on their parent atoms, with U_iso_(H) = 1.2U_eq_(C,N).

### Crystal data

**Table:**

C_11_H_11_BrN_2_O_4_	*F*(000) = 632
*M**_r_* = 315.12	*D*_x_ = 1.714 Mg m^−^^3^
Monoclinic, *P*2_1_	Mo *K*α radiation, λ = 0.71073 Å
Hall symbol: P 2yb	Cell parameters from 25 reflections
*a* = 9.4150 (19) Å	θ = 10–13°
*b* = 14.458 (3) Å	µ = 3.38 mm^−^^1^
*c* = 9.6170 (19) Å	*T* = 294 K
β = 111.14 (3)°	Block, colorless
*V* = 1221.0 (5) Å^3^	0.20 × 0.10 × 0.10 mm
*Z* = 4	

### Data collection

**Table:**

Enraf–Nonius CAD-4 diffractometer	2669 reflections with *I* > 2σ(*I*)
Radiation source: fine-focus sealed tube	*R*_int_ = 0.061
graphite	θ_max_ = 26.0°, θ_min_ = 2.3°
ω/2θ scans	*h* = 0→11
Absorption correction: ψ scan (North *et al.*, 1968)	*k* = −10→17
*T*_min_ = 0.552, *T*_max_ = 0.729	*l* = −11→11
4504 measured reflections	3 standard reflections every 120 min
4256 independent reflections	intensity decay: 1%

### Refinement

**Table:**

Refinement on *F*^2^	Secondary atom site location: difference Fourier map
Least-squares matrix: full	Hydrogen site location: inferred from neighbouring sites
*R*[*F*^2^ > 2σ(*F*^2^)] = 0.063	H-atom parameters constrained
*wR*(*F*^2^) = 0.181	*w* = 1/[σ^2^(*F*_o_^2^) + (0.1*P*)^2^ + 1.54*P*] where *P* = (*F*_o_^2^ + 2*F*_c_^2^)/3
*S* = 1.00	(Δ/σ)_max_ < 0.001
4256 reflections	Δρ_max_ = 0.54 e Å^−^^3^
325 parameters	Δρ_min_ = −0.82 e Å^−^^3^
0 restraints	Absolute structure: Flack (1983), 1758 Friedel pairs
Primary atom site location: structure-invariant direct methods	Flack parameter: 0.00 (2)

### Special details

**Table:**

Geometry. All e.s.d.'s (except the e.s.d. in the dihedral angle between two l.s. planes) are estimated using the full covariance matrix. The cell e.s.d.'s are taken into account individually in the estimation of e.s.d.'s in distances, angles and torsion angles; correlations between e.s.d.'s in cell parameters are only used when they are defined by crystal symmetry. An approximate (isotropic) treatment of cell e.s.d.'s is used for estimating e.s.d.'s involving l.s. planes.
Refinement. Refinement of *F*^2^ against ALL reflections. The weighted *R*-factor *wR* and goodness of fit *S* are based on *F*^2^, conventional *R*-factors *R* are based on *F*, with *F* set to zero for negative *F*^2^. The threshold expression of *F*^2^ > σ(*F*^2^) is used only for calculating *R*-factors(gt) *etc*. and is not relevant to the choice of reflections for refinement. *R*-factors based on *F*^2^ are statistically about twice as large as those based on *F*, and *R*- factors based on ALL data will be even larger.

### Fractional atomic coordinates and isotropic or equivalent isotropic displacement parameters (Å^2^)

**Table:**

	*x*	*y*	*z*	*U*_iso_*/*U*_eq_	
Br1	0.02596 (15)	0.99788 (7)	0.97303 (14)	0.0608 (5)	
Br2	0.51991 (15)	0.59675 (7)	0.96846 (14)	0.0584 (4)	
O1	0.2071 (7)	0.8476 (4)	1.2310 (7)	0.0342 (15)	
O2	0.3547 (9)	0.7557 (5)	1.1503 (8)	0.045 (2)	
O3	0.5492 (8)	0.9264 (5)	1.5118 (7)	0.0425 (17)	
O4	0.5063 (11)	0.6732 (6)	1.5238 (9)	0.072 (3)	
O5	0.1786 (8)	0.3598 (6)	0.8478 (8)	0.0402 (19)	
O6	0.3262 (6)	0.4292 (4)	0.7424 (6)	0.0289 (14)	
O7	−0.0743 (8)	0.2608 (5)	0.5162 (8)	0.051 (2)	
O8	−0.0128 (9)	0.5151 (6)	0.4748 (10)	0.077 (3)	
N1	0.3494 (10)	1.0032 (7)	1.3501 (9)	0.054 (2)	
H1A	0.3872	1.0569	1.3798	0.065*	
H1B	0.2618	0.9983	1.2798	0.065*	
N2	0.3894 (12)	0.6010 (7)	1.3088 (9)	0.065 (3)	
H2A	0.3936	0.5487	1.3527	0.078*	
H2B	0.3480	0.6046	1.2133	0.078*	
N3	0.1299 (12)	0.2007 (8)	0.6938 (12)	0.083 (4)	
H3B	0.1123	0.1461	0.6557	0.100*	
H3C	0.2075	0.2103	0.7737	0.100*	
N4	0.2056 (10)	0.5835 (6)	0.5937 (10)	0.058 (3)	
H4B	0.1804	0.6354	0.5475	0.070*	
H4C	0.2946	0.5768	0.6609	0.070*	
C1	−0.1253 (13)	0.8539 (9)	0.7843 (12)	0.054 (3)	
H1C	−0.1973	0.8994	0.7406	0.065*	
C2	−0.1452 (15)	0.7645 (9)	0.7283 (14)	0.063 (3)	
H2C	−0.2335	0.7490	0.6491	0.076*	
C3	−0.0347 (14)	0.6989 (9)	0.7897 (12)	0.060 (3)	
H3A	−0.0477	0.6397	0.7492	0.072*	
C4	0.0952 (12)	0.7193 (8)	0.9104 (12)	0.045 (3)	
H4A	0.1685	0.6738	0.9501	0.055*	
C5	0.1177 (10)	0.8071 (7)	0.9733 (10)	0.032 (2)	
C6	0.0023 (11)	0.8736 (7)	0.9049 (11)	0.041 (2)	
C7	0.2506 (12)	0.8300 (7)	1.1051 (11)	0.042 (3)	
H7A	0.3017	0.8846	1.0846	0.051*	
C8	0.4385 (13)	0.7664 (7)	1.3091 (12)	0.043 (3)	
H8A	0.5406	0.7913	1.3284	0.051*	
C9	0.3383 (11)	0.8363 (7)	1.3586 (10)	0.033 (2)	
H9A	0.3110	0.8091	1.4389	0.040*	
C10	0.4257 (11)	0.9281 (7)	1.4125 (10)	0.035 (2)	
C11	0.4458 (12)	0.6747 (7)	1.3868 (11)	0.040 (2)	
C12	0.6632 (12)	0.4664 (8)	1.1863 (12)	0.047 (3)	
H12A	0.7377	0.5119	1.2195	0.057*	
C13	0.6768 (12)	0.3799 (8)	1.2638 (11)	0.047 (3)	
H13A	0.7629	0.3679	1.3475	0.056*	
C14	0.5657 (12)	0.3166 (9)	1.2157 (12)	0.050 (3)	
H14A	0.5721	0.2619	1.2687	0.060*	
C15	0.4429 (12)	0.3328 (7)	1.0882 (11)	0.042 (2)	
H15A	0.3687	0.2872	1.0542	0.051*	
C16	0.4242 (11)	0.4149 (7)	1.0072 (10)	0.038 (2)	
C17	0.5378 (11)	0.4786 (7)	1.0634 (10)	0.038 (2)	
C18	0.2854 (10)	0.4319 (7)	0.8716 (10)	0.035 (2)	
H18A	0.2392	0.4915	0.8789	0.042*	
C19	0.0858 (10)	0.3643 (7)	0.6949 (11)	0.033 (2)	
H19A	−0.0053	0.4007	0.6842	0.040*	
C20	0.1849 (12)	0.4189 (7)	0.6234 (10)	0.041 (3)	
H20A	0.1984	0.3824	0.5433	0.049*	
C21	0.0373 (12)	0.2699 (7)	0.6293 (12)	0.041 (3)	
C22	0.1035 (12)	0.5114 (7)	0.5599 (10)	0.039 (2)	

### Atomic displacement parameters (Å^2^)

**Table:**

	*U*^11^	*U*^22^	*U*^33^	*U*^12^	*U*^13^	*U*^23^
Br1	0.0828 (10)	0.0392 (9)	0.0483 (8)	0.0150 (7)	0.0090 (7)	0.0055 (7)
Br2	0.0742 (9)	0.0320 (8)	0.0488 (8)	−0.0102 (7)	−0.0022 (7)	0.0053 (7)
O1	0.041 (3)	0.031 (4)	0.028 (4)	0.001 (3)	0.009 (3)	−0.002 (3)
O2	0.070 (5)	0.033 (5)	0.028 (4)	0.010 (4)	0.011 (4)	−0.001 (3)
O3	0.061 (4)	0.026 (4)	0.031 (4)	−0.005 (3)	0.006 (3)	0.001 (3)
O4	0.113 (7)	0.032 (5)	0.035 (4)	0.013 (4)	−0.016 (4)	−0.006 (4)
O5	0.048 (4)	0.043 (5)	0.023 (4)	−0.007 (4)	0.005 (3)	0.000 (3)
O6	0.034 (3)	0.031 (4)	0.014 (3)	0.007 (3)	−0.001 (2)	0.000 (3)
O7	0.060 (4)	0.034 (5)	0.032 (4)	−0.001 (3)	−0.016 (3)	−0.005 (3)
O8	0.073 (5)	0.043 (6)	0.068 (6)	−0.003 (4)	−0.031 (5)	0.019 (4)
N1	0.063 (5)	0.030 (5)	0.045 (5)	−0.002 (5)	−0.010 (4)	−0.011 (5)
N2	0.121 (8)	0.030 (5)	0.024 (5)	−0.022 (6)	0.001 (5)	0.002 (5)
N3	0.091 (7)	0.033 (6)	0.071 (7)	0.008 (6)	−0.038 (6)	−0.003 (6)
N4	0.054 (5)	0.042 (7)	0.056 (6)	−0.003 (5)	−0.007 (4)	0.023 (5)
C1	0.060 (5)	0.062 (6)	0.035 (5)	0.007 (5)	0.012 (4)	0.007 (5)
C2	0.071 (6)	0.059 (6)	0.046 (6)	−0.016 (5)	0.006 (5)	0.006 (5)
C3	0.086 (6)	0.048 (6)	0.040 (5)	−0.012 (5)	0.015 (4)	−0.010 (5)
C4	0.062 (5)	0.032 (5)	0.037 (5)	−0.003 (4)	0.012 (4)	0.001 (4)
C5	0.034 (4)	0.033 (5)	0.029 (4)	−0.007 (4)	0.012 (3)	0.006 (4)
C6	0.054 (5)	0.038 (5)	0.030 (5)	0.006 (4)	0.015 (4)	0.008 (4)
C7	0.067 (7)	0.018 (6)	0.038 (6)	0.004 (5)	0.016 (5)	0.001 (5)
C8	0.064 (7)	0.015 (6)	0.033 (6)	0.001 (5)	−0.002 (5)	−0.003 (5)
C9	0.052 (6)	0.027 (6)	0.022 (5)	0.004 (4)	0.015 (4)	0.007 (4)
C10	0.052 (6)	0.033 (7)	0.019 (5)	0.004 (5)	0.010 (4)	0.002 (4)
C11	0.058 (6)	0.024 (6)	0.025 (5)	0.007 (5)	0.000 (4)	−0.005 (4)
C12	0.053 (5)	0.044 (6)	0.040 (5)	0.002 (4)	0.009 (4)	−0.003 (4)
C13	0.052 (5)	0.049 (6)	0.030 (5)	0.008 (4)	0.004 (4)	0.001 (4)
C14	0.058 (5)	0.050 (6)	0.034 (5)	0.004 (4)	0.008 (4)	0.007 (4)
C15	0.062 (5)	0.025 (5)	0.031 (5)	0.001 (4)	0.007 (4)	−0.003 (4)
C16	0.058 (5)	0.027 (5)	0.023 (4)	0.004 (4)	0.009 (4)	−0.003 (4)
C17	0.055 (5)	0.021 (5)	0.031 (5)	0.003 (4)	0.006 (4)	−0.004 (4)
C18	0.049 (5)	0.031 (6)	0.022 (5)	−0.006 (5)	0.009 (4)	−0.003 (4)
C19	0.033 (5)	0.025 (6)	0.036 (6)	−0.005 (4)	0.008 (4)	−0.007 (5)
C20	0.071 (7)	0.021 (6)	0.024 (5)	0.020 (5)	0.009 (5)	0.000 (4)
C21	0.048 (6)	0.028 (6)	0.038 (6)	−0.001 (5)	0.003 (5)	0.007 (5)
C22	0.059 (6)	0.030 (7)	0.019 (5)	0.003 (5)	0.002 (4)	0.005 (4)

### Geometric parameters (Å, °)

**Table:**

Br1—C6	1.897 (11)	C2—H2C	0.9300
Br2—C17	1.915 (10)	C3—C4	1.381 (15)
O1—C9	1.402 (11)	C3—H3A	0.9300
O1—C7	1.434 (11)	C4—C5	1.389 (15)
O2—C7	1.414 (12)	C4—H4A	0.9300
O2—C8	1.453 (12)	C5—C6	1.422 (13)
O3—C10	1.210 (11)	C5—C7	1.462 (14)
O4—C11	1.232 (12)	C7—H7A	0.9800
O5—C18	1.409 (11)	C8—C11	1.511 (14)
O5—C19	1.416 (11)	C8—C9	1.568 (14)
O6—C20	1.416 (11)	C8—H8A	0.9800
O6—C18	1.427 (11)	C9—C10	1.549 (14)
O7—C21	1.217 (11)	C9—H9A	0.9800
O8—C22	1.107 (11)	C12—C17	1.348 (13)
N1—C10	1.321 (12)	C12—C13	1.437 (16)
N1—H1A	0.8600	C12—H12A	0.9300
N1—H1B	0.8600	C13—C14	1.341 (15)
N2—C11	1.301 (13)	C13—H13A	0.9300
N2—H2A	0.8600	C14—C15	1.369 (14)
N2—H2B	0.8600	C14—H14A	0.9300
N3—C21	1.324 (13)	C15—C16	1.395 (14)
N3—H3B	0.8600	C15—H15A	0.9300
N3—H3C	0.8600	C16—C17	1.367 (14)
N4—C22	1.375 (13)	C16—C18	1.497 (12)
N4—H4B	0.8600	C18—H18A	0.9800
N4—H4C	0.8600	C19—C21	1.504 (14)
C1—C6	1.366 (14)	C19—C20	1.559 (15)
C1—C2	1.386 (18)	C19—H19A	0.9800
C1—H1C	0.9300	C20—C22	1.552 (13)
C2—C3	1.374 (17)	C20—H20A	0.9800
			
C9—O1—C7	106.8 (7)	C10—C9—H9A	109.8
C7—O2—C8	107.2 (8)	C8—C9—H9A	109.8
C18—O5—C19	105.7 (7)	O3—C10—N1	125.8 (9)
C20—O6—C18	103.7 (7)	O3—C10—C9	119.4 (8)
C10—N1—H1A	120.0	N1—C10—C9	114.6 (8)
C10—N1—H1B	120.0	O4—C11—N2	122.5 (10)
H1A—N1—H1B	120.0	O4—C11—C8	117.7 (9)
C11—N2—H2A	120.0	N2—C11—C8	119.8 (9)
C11—N2—H2B	120.0	C17—C12—C13	117.1 (10)
H2A—N2—H2B	120.0	C17—C12—H12A	121.5
C21—N3—H3B	120.0	C13—C12—H12A	121.5
C21—N3—H3C	120.0	C14—C13—C12	120.2 (10)
H3B—N3—H3C	120.0	C14—C13—H13A	119.9
C22—N4—H4B	120.0	C12—C13—H13A	119.9
C22—N4—H4C	120.0	C13—C14—C15	119.6 (11)
H4B—N4—H4C	120.0	C13—C14—H14A	120.2
C6—C1—C2	118.7 (12)	C15—C14—H14A	120.2
C6—C1—H1C	120.7	C14—C15—C16	122.8 (11)
C2—C1—H1C	120.7	C14—C15—H15A	118.6
C3—C2—C1	120.1 (12)	C16—C15—H15A	118.6
C3—C2—H2C	120.0	C17—C16—C15	115.4 (9)
C1—C2—H2C	120.0	C17—C16—C18	123.1 (9)
C2—C3—C4	121.2 (12)	C15—C16—C18	121.4 (9)
C2—C3—H3A	119.4	C12—C17—C16	124.8 (10)
C4—C3—H3A	119.4	C12—C17—Br2	115.7 (8)
C3—C4—C5	120.7 (11)	C16—C17—Br2	119.4 (7)
C3—C4—H4A	119.6	O5—C18—O6	103.9 (7)
C5—C4—H4A	119.6	O5—C18—C16	111.5 (8)
C4—C5—C6	116.5 (9)	O6—C18—C16	109.3 (8)
C4—C5—C7	122.2 (9)	O5—C18—H18A	110.7
C6—C5—C7	121.3 (9)	O6—C18—H18A	110.7
C1—C6—C5	122.8 (11)	C16—C18—H18A	110.7
C1—C6—Br1	116.7 (8)	O5—C19—C21	112.0 (9)
C5—C6—Br1	120.4 (8)	O5—C19—C20	103.8 (7)
O2—C7—O1	104.7 (8)	C21—C19—C20	114.5 (9)
O2—C7—C5	112.1 (8)	O5—C19—H19A	108.8
O1—C7—C5	110.9 (8)	C21—C19—H19A	108.8
O2—C7—H7A	109.7	C20—C19—H19A	108.8
O1—C7—H7A	109.7	O6—C20—C22	114.5 (8)
C5—C7—H7A	109.7	O6—C20—C19	103.5 (7)
O2—C8—C11	109.7 (8)	C22—C20—C19	108.8 (8)
O2—C8—C9	103.3 (8)	O6—C20—H20A	110.0
C11—C8—C9	109.9 (9)	C22—C20—H20A	110.0
O2—C8—H8A	111.2	C19—C20—H20A	110.0
C11—C8—H8A	111.2	O7—C21—N3	123.2 (10)
C9—C8—H8A	111.2	O7—C21—C19	120.4 (9)
O1—C9—C10	112.7 (8)	N3—C21—C19	116.1 (9)
O1—C9—C8	104.3 (7)	O8—C22—N4	124.0 (10)
C10—C9—C8	110.4 (8)	O8—C22—C20	123.2 (10)
O1—C9—H9A	109.8	N4—C22—C20	110.9 (8)
			
C6—C1—C2—C3	−2.9 (18)	C17—C12—C13—C14	−1.8 (16)
C1—C2—C3—C4	2.1 (19)	C12—C13—C14—C15	3.3 (17)
C2—C3—C4—C5	0.0 (19)	C13—C14—C15—C16	−2.5 (18)
C3—C4—C5—C6	−1.2 (15)	C14—C15—C16—C17	0.3 (16)
C3—C4—C5—C7	177.3 (10)	C14—C15—C16—C18	−177.5 (10)
C2—C1—C6—C5	1.7 (16)	C13—C12—C17—C16	−0.5 (17)
C2—C1—C6—Br1	178.3 (9)	C13—C12—C17—Br2	177.4 (8)
C4—C5—C6—C1	0.4 (15)	C15—C16—C17—C12	1.2 (16)
C7—C5—C6—C1	−178.2 (10)	C18—C16—C17—C12	179.0 (10)
C4—C5—C6—Br1	−176.1 (8)	C15—C16—C17—Br2	−176.6 (7)
C7—C5—C6—Br1	5.3 (13)	C18—C16—C17—Br2	1.2 (14)
C8—O2—C7—O1	−33.6 (10)	C19—O5—C18—O6	−40.6 (9)
C8—O2—C7—C5	−153.9 (9)	C19—O5—C18—C16	−158.2 (8)
C9—O1—C7—O2	38.4 (9)	C20—O6—C18—O5	44.5 (8)
C9—O1—C7—C5	159.5 (8)	C20—O6—C18—C16	163.6 (8)
C4—C5—C7—O2	4.6 (14)	C17—C16—C18—O5	−173.1 (9)
C6—C5—C7—O2	−176.9 (8)	C15—C16—C18—O5	4.5 (14)
C4—C5—C7—O1	−111.9 (10)	C17—C16—C18—O6	72.7 (12)
C6—C5—C7—O1	66.6 (11)	C15—C16—C18—O6	−109.7 (10)
C7—O2—C8—C11	133.8 (9)	C18—O5—C19—C21	144.8 (9)
C7—O2—C8—C9	16.6 (10)	C18—O5—C19—C20	20.7 (10)
C7—O1—C9—C10	92.9 (9)	C18—O6—C20—C22	88.1 (9)
C7—O1—C9—C8	−26.9 (9)	C18—O6—C20—C19	−30.1 (9)
O2—C8—C9—O1	6.4 (10)	O5—C19—C20—O6	6.1 (9)
C11—C8—C9—O1	−110.6 (8)	C21—C19—C20—O6	−116.3 (8)
O2—C8—C9—C10	−114.9 (9)	O5—C19—C20—C22	−116.0 (8)
C11—C8—C9—C10	128.1 (9)	C21—C19—C20—C22	121.6 (9)
O1—C9—C10—O3	−172.2 (8)	O5—C19—C21—O7	158.6 (10)
C8—C9—C10—O3	−56.0 (11)	C20—C19—C21—O7	−83.5 (13)
O1—C9—C10—N1	12.7 (12)	O5—C19—C21—N3	−27.2 (14)
C8—C9—C10—N1	128.9 (9)	C20—C19—C21—N3	90.7 (13)
O2—C8—C11—O4	−176.2 (9)	O6—C20—C22—O8	−173.5 (11)
C9—C8—C11—O4	−63.3 (13)	C19—C20—C22—O8	−58.3 (14)
O2—C8—C11—N2	3.9 (15)	O6—C20—C22—N4	21.5 (12)
C9—C8—C11—N2	116.9 (11)	C19—C20—C22—N4	136.7 (9)

### Hydrogen-bond geometry (Å, °)

**Table:**

*D*—H···*A*	*D*—H	H···*A*	*D*···*A*	*D*—H···*A*
N1—H1B···O1	0.86	2.25	2.657 (12)	109
N2—H2B···O2	0.86	2.27	2.660 (12)	107
N3—H3B···O8^i^	0.86	2.28	3.123 (14)	167
N3—H3C···O5	0.86	2.32	2.684 (14)	106
N4—H4B···O7^ii^	0.86	2.05	2.876 (11)	160
N4—H4C···O6	0.86	2.26	2.672 (11)	110

Symmetry codes: (i) −*x*, *y*−1/2, −*z*+1; (ii) −*x*, *y*+1/2, −*z*+1.
